# Age Differences in the Relationship Between Interoception and Emotional Processing

**DOI:** 10.3390/bs16050672

**Published:** 2026-04-29

**Authors:** Sophie Cawkwell, Kata Pauly-Takacs, Katerina Zoe Kolokotroni, Gaby Pfeifer

**Affiliations:** 1 School of Humanities and Social Sciences, Leeds Beckett University, Leeds LS1 3HE, UKk.pauly-takacs@leedsbeckett.ac.uk (K.P.-T.); z.kolokotroni@leedsbeckett.ac.uk (K.Z.K.); 2 School of Psychology, Faculty of Environmental and Life Sciences, University of Southampton, Southampton SO17 1BJ, UK

**Keywords:** interoception, emotional processing, age differences, associative memory, mind–body connection, positivity bias, Socioemotional Selectivity Theory

## Abstract

Understanding how bodily signals shape emotional cognition across adulthood is critical for explaining age-related changes in emotional learning and memory. This study investigated age-related differences in interoceptive sensitivity and emotional associative memory. Interoceptive sensitivity was used as an umbrella term to refer to sensitivity to internal bodily signals across interoceptive accuracy, attention, beliefs, and insight, while emotional associative memory was defined as the ability to learn and remember emotional face–name associations. Forty younger (18–39 years) and forty older (60–85 years) adults completed behavioural and self-report interoceptive measures alongside an emotional face–name learning, recall, and recognition paradigm. No significant age differences emerged for interoceptive accuracy, attention, or insight. However, older adults reported greater trust in, and less worry about, bodily sensations, indicating selective changes in interoceptive beliefs. Older adults also showed a robust positivity bias, learning, recalling, and recognising happy face–name pairs more accurately and faster than angry or neutral pairs, whereas younger adults showed uniform performance across emotional conditions. Interoception–emotion relationships differed by age: Young adults’ interoceptive attention was positively associated with learning neutral pairs, while older adults’ interoceptive accuracy correlated with better encoding and recall of angry pairs. These findings demonstrate that age-related differences in emotional associative memory are partly rooted in changes to interoceptive processing and extend Socioemotional Selectivity Theory by identifying interoception as a physiological contributor to the positivity bias in ageing.

## 1. Introduction

Interoception, the perception of internal bodily signals, plays a fundamental role in the processing of emotions. Ageing alters how these internal signals are detected and integrated, with systematic changes emerging across physiological, subjective, and behavioural levels (Pfeifer & Cawkwell, 2025). Interoception encompasses multiple dimensions, including interoceptive accuracy (IAcc), attention (IA), beliefs (IB), and insight (II), each reflecting distinct aspects of how individuals perceive and interpret physiological states (Suksasilp & Garfinkel, 2022).

These dimensions are commonly assessed using behavioural tasks and self-report measures. Interoceptive accuracy, which refers to the ability to detect internal bodily signals such as heartbeats, is typically measured through tasks such as the Heartbeat Counting Task (HCT; Schandry, 1981) and the Heartbeat Discrimination Task (HDT; Whitehead et al., 1977). Interoceptive attention and beliefs, which reflect how individuals attend to and interpret bodily sensations, are usually captured through questionnaires such as the Body Perception Questionnaire (BPQ; Cabrera et al., 2018) and the Multidimensional Assessment of Interoceptive Awareness, Version 2 (MAIA-2; Mehling et al., 2018). Interoceptive insight, a metacognitive dimension, combines behavioural performance with confidence ratings to assess individuals’ awareness of their own IAcc (Garfinkel et al., 2015). With ageing, these dimensions do not change in parallel; behavioural accuracy, subjective beliefs, and metacognitive insight show divergent age-related trajectories (Pfeifer & Cawkwell, 2025).

Foundational emotion theories (James, 1884; Lange, 1885) suggest that emotions emerge from the perception of physiological changes. These changes, such as fluctuations in heart rate or respiration, are mediated by the autonomic nervous system and interpreted through interoceptive processes (Craig, 2002; Ueno et al., 2025). Interoception therefore plays a central role in generating affective states and shaping self-awareness (Sel, 2014; Monti et al., 2022). Brain regions such as the insular cortex and anterior cingulate cortex support this mind–body integration, combining somatic and external signals to construct emotional states (Critchley et al., 2004; Terasawa et al., 2013).

A body of empirical work shows that interoceptive dimensions are largely dissociable, with low correlations across behavioural, self-report, and metacognitive measures (Calì et al., 2015; Garfinkel et al., 2015, 2016a; Murphy et al., 2019; Pearson & Pfeifer, 2022; Suksasilp & Garfinkel, 2022). Nonetheless, selective interdependencies do occur. Physiological research, predominantly with younger adults, demonstrates that afferent cardiac signals can preconsciously influence cognition and emotion. Emotional and mnemonic processing varies systematically with the timing of stimuli across the cardiac cycle, and these effects interact with individual differences in interoceptive sensitivity. For example, word memory is enhanced when stimuli are encoded during systole, particularly among individuals with higher IAcc and II (Garfinkel et al., 2013). Emotional learning and memory for negative stimuli, such as fear or threat, also improve when stimuli coincide with systolic cardiac signals, highlighting the role of visceral afferents in shaping cognitive–emotional processes (Pfeifer et al., 2017; Garfinkel et al., 2020). Complementing this, neurophysiological findings show that subliminal anger cues elicit increases in systolic blood pressure, and the magnitude of these vascular responses predicts subsequent slowing in semantic decision-making, indicating that afferent cardiovascular signals can bias cognition outside conscious awareness (Garfinkel et al., 2016b).

A further factor relevant to interoceptive functioning across adulthood is Body Mass Index (BMI). Research demonstrates that higher BMI is consistently associated with reduced interoceptive ability in adults, with meta-analytic evidence showing small but reliable deficits across behavioural and self-report measures of interoception (Robinson et al., 2021; Willem et al., 2019). Importantly, these effects do not appear to be specific to any single interoceptive dimension, suggesting that elevated BMI may influence cardiac interoceptive accuracy, subjective beliefs, and metacognitive insight to a similar degree. Evidence from lifespan research further indicates that BMI may partly mediate age-related declines in cardiac interoceptive accuracy: ageing is associated with increases in BMI, which in turn predict lower accuracy on heartbeat-based tasks (Murphy et al., 2018). These findings imply that BMI-related physiological changes could compound age-related reductions in visceral signal precision, thereby shaping interoceptive outcomes across multiple measures in later life.

With advancing age, physiological and neural changes degrade the precision of visceral inputs, weakening the moment-to-moment coupling between bodily signals and emotional experience. This biological account aligns with the concept of maturational dualism (Mendes, 2010), which proposes that the mind–body connection weakens with age, resulting in a decoupling of emotional appraisal from interoceptive sensitivity.

Given its central role in emotion, examining how interoception changes with age is crucial for understanding age-related differences in emotional processing (Pfeifer & Cawkwell, 2025). Current findings in this area are inconsistent. Some studies report age-related declines in interoceptive accuracy (Murphy et al., 2018; Nusser et al., 2020), while others find no significant differences (Haustein et al., 2023; Ueno et al., 2020) or even report improvements in older adults (Cioffi et al., 2017). These discrepancies may stem from methodological limitations, particularly with the HCT, which has been criticised for relying on estimation rather than the true perception of heartbeats (Ring & Brener, 1996; Desmedt & Van den Bergh, 2024). They may also reflect greater individual variability among older adults, with some remaining highly attuned to bodily signals through embodied learning over time (Ross & Gillett, 2019).

Self-reported interoception also shows mixed age-related patterns. Declines in interoceptive attention have been reported using the BPQ (Murphy et al., 2018; Ulus & Aisenberg-Shafran, 2022), but findings on interoceptive beliefs are more nuanced. When assessed using the MAIA-2, age-related declines appear to be dimension-specific. For example, Dobrushina et al. (2024) found that trust in interoceptive sensations declined across adulthood, while Elliot and Pfeifer (2022) reported reduced listening to bodily sensations with age. By contrast, Dobrushina et al. (2020), using the original MAIA (Mehling et al., 2012), found no significant age-related differences in interoceptive beliefs.

Notably, while Nusser et al. (2020) found a general age-related decline in IAcc, they also observed that older adults who retained higher accuracy showed greater II, reflected in the correspondence between confidence ratings and actual performance on interoceptive tasks. This pattern implies that older adults who retain IAcc may also develop better metacognitive bodily awareness. One way to explain age-related variations in interoceptive dimensions is through the diverse physiological changes individuals experience over time, with sensations such as heart rate fluctuations, hunger, or pain taking on new meanings through decades of embodied learning. For instance, persistent pain was shown to reduce interoceptive accuracy while enhancing interoceptive beliefs (Horseburgh et al., 2024), illustrating how bodily experiences can shape interoceptive processes in different ways. These accumulated experiences may enhance some older adults’ ability to interpret and regulate interoceptive signals, and support stronger II alongside IAcc.

Interoceptive changes may help explain age-related differences in emotional experience. Older adults often report reduced emotional reactivity and increased emotional wellbeing, potentially due to diminished visceral arousal and a shift toward cognitive construction of emotions (MacCormack et al., 2021, 2022; Barrett, 2017). High-arousal emotions such as anger depend more heavily on interoceptive feedback, whereas low-arousal emotions such as sadness rely less on bodily signals and are therefore experienced more consistently across the lifespan (Sands & Isaacowitz, 2017; Scheibe et al., 2013). As visceral sensitivity declines, older adults may rely more on prior emotional knowledge and less on bodily cues, leading to altered emotional processing. Indeed, the link between interoceptive sensations and emotional experience appears stronger in young adulthood, while older adults increasingly depend on cognitively constructed emotions (MacCormack et al., 2021). These patterns are consistent with the Physiological Hypothesis of Emotional Ageing (PHEA), which argues that reduced visceral signal precision in later life increases reliance on cognitive–emotional pathways (MacCormack et al., 2022).

This shift is also reflected in memory performance, particularly for emotional information. Older adults often show reduced accuracy in learning and recognising negative stimuli (Charles et al., 2003; Grady et al., 2007). From a purely cognitive perspective, Socioemotional Selectivity Theory (SST; Carstensen, 1993, 2006) proposes that, as perceived future time becomes limited, older adults become increasingly motivated to prioritise emotionally meaningful and rewarding experiences. This motivational reorientation leads to a so-called positivity bias, whereby attention, encoding, and memory are preferentially allocated to positive rather than negative stimuli (Mather & Carstensen, 2005; Barber et al., 2016). However, this pattern of emotional selectivity may not be solely cognitive in origin. Age-related changes in interoception and visceral signal precision may shape the emotional signals on which motivational goals operate. Reduced sensitivity to high-arousal interoceptive cues may attenuate the salience of negatively valenced stimuli, which typically rely more heavily on bodily arousal for their impact (Garfinkel & Critchley, 2016; Garfinkel et al., 2013, 2020; Pfeifer et al., 2017). As a result, older adults may experience negative information as less physiologically compelling, thereby facilitating the preferential processing of emotionally rewarding, lower-arousal information. In this way, motivational shifts described by SST may be supported, and partially constrained, by underlying changes in interoceptive–affective coupling. Rather than viewing SST and biological accounts of emotional ageing as competing explanations, interoceptive change may provide an embodied mechanism through which age-related motivational priorities are expressed. As visceral input becomes noisier or less precise, emotional experience may increasingly depend on cognitively constructed meanings and prior knowledge (Barrett, 2017; MacCormack et al., 2021). This shift may amplify goal-directed emotion regulation strategies emphasised by SST, contributing to reduced emotional reactivity, enhanced wellbeing, and the characteristic positivity bias observed in later life.

Associative memory tasks further highlight age-related differences. Older adults experience difficulty in binding dissimilar pieces of information, such as faces and names, due to poorer strategic associative processes (Naveh-Benjamin, 2000; Naveh-Benjamin et al., 2009). In particular, Naveh-Benjamin et al. (2009) showed that age-related deficits in intentional face–name learning arise from reduced use of effective associative strategies, rather than from general memory decline. These associative difficulties are further compounded by reduced neural efficiency (Park et al., 2012; Pfeifer et al., 2014, 2016, 2019). Across these studies, ageing is characterised by neural broadening, whereby neural responses become less selective and more diffuse. This reduced specificity means that older adults engage a wider set of neural resources to support the same processing demands, reflecting compensatory recruitment rather than efficient encoding. As a result, associative binding becomes more effortful and less precise, contributing to the disproportionate challenges older adults face when learning and remembering face–name pairings. Older adults face further constraints due to slower processing speeds (Salthouse, 1996), which interfere with the timely coordination of encoding operations required for successful associative binding (Salthouse, 1996). Slower processing limits the ability to simultaneously maintain, organise, and integrate face and name information, increasing the likelihood that key associative links are lost before they can be fully encoded.

Adding emotional content may selectively enhance memory for positive stimuli in older adults but not necessarily for negative ones (Charles et al., 2003). In Charles et al.’s study, older adults recalled fewer negative images than younger adults, indicating an age-related reduction in memory for negatively valenced material relative to positive and neutral content, a pattern consistent with SST.

While affective stimuli typically improve memory for individual items (Keightley et al., 2011; Righi et al., 2012), in associative learning contexts, attentional narrowing (Easterbrook, 1959) suggests that high-arousal emotions may focus attention on the emotional stimulus itself, thereby reducing attention to peripheral details such as names. This mechanism may impair associative memory for negative stimuli (Rimmele et al., 2011), particularly in younger adults who are more physiologically reactive. By contrast, older adults, who exhibit reduced visceral arousal and a motivational preference for positive information, may show selective improvements in learning and remembering happy face–name pairs, while continuing to struggle with negative or neutral associations. This pattern reflects both a cognitive adaptation and a biological shift in emotional processing across the lifespan.

Together, these frameworks indicate that age-related changes in emotional processing reflect an interaction between diminished physiological reactivity and increasing reliance on cognitive regulation aligned with wellbeing goals. Understanding emotional ageing therefore requires an integrated approach that considers how age-related changes in interoception interact with cognitive and motivational processes to shape emotional experience.

### The Present Study

This study investigated age-related differences in interoception and emotional processing by comparing young (18–39) and older (60–85) adults. Four dimensions of interoception were assessed: accuracy (HCT, HDT), attention (BPQ-VSF), beliefs (MAIA-2), and insight (confidence ratings corresponding with accuracy). Based on prior research, we hypothesised that young adults would exhibit greater interoceptive accuracy and attention, whereas older adults would demonstrate stronger interoceptive insight. Given the variability in prior findings across MAIA and MAIA-2 subscales, our hypotheses for interoceptive beliefs were largely exploratory. Age differences in interoceptive beliefs were expected to vary across MAIA-2 subscales, reflecting the heterogeneous findings in the literature. However, we expected that older adults would score lower on the Noticing subscale, as this dimension most closely aligns with interoceptive attention as measured by the BPQ (see Mehling et al., 2012, p. 3), for which age-related declines have been consistently documented.

Emotional processing was examined using a face–name associative learning paradigm incorporating emotional expressions (angry, neutral, happy) to assess learning, recall, and recognition. Recall and recognition were included because they index complementary memory processes: recall relies primarily on recollection, whereas recognition can be supported by both recollection and familiarity. These processes show distinct behavioural and neural profiles (Yonelinas et al., 2024; Nie & Wu, 2023) and are differentially affected by ageing, with older adults typically showing greater declines in recollection-based recall but relatively preserved familiarity-based recognition (Naveh-Benjamin et al., 2009; Grady et al., 2003). Including both measures therefore provides a more complete characterisation of age-related differences in emotional associative memory.

Consistent with the well-established positivity bias in ageing, older adults were expected to show enhanced learning and memory for happy face–name pairs. By contrast, younger adults were expected to perform consistently across emotional categories; however, due to attentional narrowing toward emotionally salient, threat-related stimuli, they may exhibit reduced performance for angry face–name associations.

Finally, we explored whether interoception and emotional processing were associated across age groups. We expected this relationship to be stronger in young adults, with interoceptive sensitivity more strongly associated with emotional associative learning and memory.

## 2. Materials and Methods

### 2.1. Participants

Eighty participants were included: 40 younger adults (18–39 years, M = 23.03, SD = 6.12) and 40 older adults (60–77 years, M = 68.48, SD = 5.72), matched for sex (50:50 female:male). Recruitment was conducted via university platforms (SONA, posters), social media (Facebook), and community organisations such as the University of the Third Age (U3A). Eligibility criteria included UK residency (≥3 months), English fluency, normal/corrected hearing, and absence of cardiac, neurological, or recent mental health conditions. Inhaler-controlled asthma was permitted. These criteria were implemented as heart conditions such as arrhythmia can cause ambiguity when undertaking interoceptive tasks (Khalsa et al., 2009a), and mental health conditions (e.g., anxiety) have been found to lead to atypical interoceptive interpretations (Paulus & Stein, 2010; Quadt et al., 2018). None of the participants was on antihypertensive medication. Participants taking statins were eligible to participate because these treatments generally do not alter cardiac rhythm or autonomic feedback in ways that would affect interoceptive performance, particularly in the absence of diagnosed cardiac conditions (Kostapanos et al., 2007).

Older adults completed the Mini-Mental State Examination (MMSE; Folstein et al., 1975), with scores ≥25 required for inclusion. An a priori power analysis using G*Power 3.1 determined that a minimum total sample size of 64 participants was required (effect size: f = 0.40, power: 0.80). Of 103 screened, 86 completed the study; 6 were excluded due to being extreme outliers or task non-compliance.

Most participants were UK nationals who identified as White British. Ethnic diversity was more pronounced among younger adults, including individuals from Asian, Black, and Mixed/Multiple ethnic backgrounds.

### 2.2. Materials

A combination of subjective questionnaires, physiological tasks, and experimental paradigms was used to assess interoception and emotional associative memory.

### 2.3. Interoceptive Measures

Interoceptive Attention: Assessed using the Very Short Form of the Body Perception Questionnaire (BPQ-VSF; Cabrera et al., 2018), a 12-item scale measuring awareness of bodily sensations (e.g., “How hard my heart is beating”). Responses were rated on a 5-point Likert scale (1 = Never, 5 = Always). Scores were averaged to produce a mean interoceptive attention score. Internal consistency was high across age groups (see Table 1).

Interoceptive Beliefs: Measured using the Multidimensional Assessment of Interoceptive Awareness, Version 2 (MAIA-2; Mehling et al., 2018), a 37-item self-report questionnaire comprising eight subscales: Noticing (4 items), Not-Distracting (6 items), Not-Worrying (5 items), Attention Regulation (7 items), Emotional Awareness (5 items), Self-Regulation (4 items), Body Listening (3 items), and Trusting (3 items). Participants rate each item on a 6-point scale (0 = Never to 5 = Always), and subscale scores are computed as the average of their respective items. The questionnaire takes approximately 15 min to complete. Reliability in the present sample was moderate to strong across subscales, although the ‘Noticing’ subscale fell below 0.70 for younger adults (see Table 1).

The HCT (Schandry, 1981) required participants to silently count their heartbeats without physically checking their pulse. Each participant completed six trials, with time intervals randomly presented at 25, 30, 35, 40, 45, and 50 s (following Garfinkel et al., 2015). At the end of each interval, they reported the number of heartbeats they perceived. Accuracy for each trial was calculated using the following formula: 1 − (|nbeats_real_ − nbeats_reported_|)/((nbeats_real_ + nbeats_reported_)/2). This formula provides a normalised index of accuracy, accounting for both over- and underestimation. A mean accuracy score was computed across all six trials.

Interoceptive Accuracy (IAcc): Interoceptive accuracy was assessed using two tasks: the Heartbeat Counting Task (HCT) and the Heartbeat Discrimination Task (HDT). During these tasks, participants were asked to wear a reusable soft-sensor pulse oximeter (XPOD^®^ 3012 LP with USB Connector; Nonin Medical, Inc., Plymouth, MN, USA) on their left index finger to measure heartbeats. Participants’ left hand and forearm were elevated on a cushion to further minimise tactile feedback. Reliability in the present sample was strong for the HCT but comparatively weak for the HDT, falling below 0.60, consistent with reported concerns regarding the psychometric properties of the latter task (Brener & Ring, 2016) (see Table 1).

In the HDT (Whitehead et al., 1977), participants listened to a series of ten consecutive auditory tones (each lasting 100 ms) and judged whether the tones were presented in synchrony with their own heartbeat. Each participant completed 20 trials (10 synchronous and 10 asynchronous), preceded by a single practice trial. To account for pulse transit time from the heart to the index finger, tones in the synchronous condition were delayed by 250 ms after the R-wave. In the asynchronous condition, tones were delayed by 550 ms, introducing an additional 300 ms beyond the estimated transit time. After each trial, participants indicated whether they perceived the tones to be in sync with their heartbeat. Accuracy was calculated as the mean proportion of correct judgments across all 20 trials.

Interoceptive insight: Interoceptive insight reflects the correspondence between participants’ confidence ratings and the accuracy of their interoceptive performance. Following each trial of the respective IAcc tasks (N_trials_ = 6 for the HCT; N_trials_ = 20 for the HDT), participants rated their confidence in the accuracy of their responses. Confidence was measured using a 10 cm Visual Analogue Scale (VAS), on which participants marked their perceived confidence by drawing a line across the scale. A score of 0 indicated “Not Confident At All” (no heartbeat awareness), while a score of 10 indicated “Very Confident” (full awareness of heartbeat). Interoceptive insight scores were computed by quantifying the correspondence between confidence and behavioural accuracy, as detailed in Section 3.3.

### 2.4. Emotional Associative Learning and Memory

Emotional associative learning: Participants completed a face–name associative learning task adapted from Pfeifer et al. (2017), designed to engage trial-and-error learning. They were presented with 12 face–name pairs (6 male, 6 female), each displaying one of three emotional expressions: angry, neutral, or happy. Faces were sourced from the FACES database (Ebner et al., 2010), which provides picture-specific normative ratings for distinctiveness and facial expression based on validation by 154 raters, ensuring these dimensions were controlled at the database level. The faces were age-matched to participant groups (young adults viewed only young faces and older adults viewed only older faces) to account for own-age bias in facial recognition, whereby individuals show better memory for faces from their own age group (Fulton & Bartlett, 1991; Rhodes & Anastasi, 2012). Using age-congruent faces ensured that participants evaluated emotional expressions within their own age range, reducing the likelihood that emotional expressions were perceived with differing intensity across participant age groups and thereby improving comparability. Names were selected from the Social Security Administration’s top 200 names from relevant birth decades (1930s–1960s for older adults; 1980s–2000s for younger adults, (https://www.ssa.gov/oact/babynames/decades/index.html, (accessed on 29 October 2025)) and matched for length and frequency.

**Figure 1:**
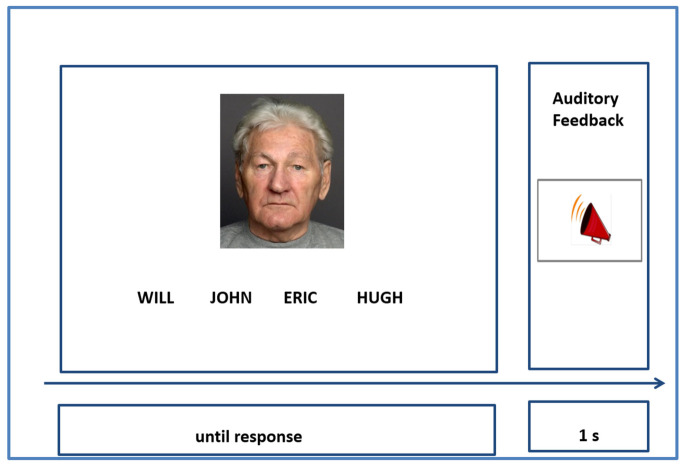
Example of an Associative Learning Trial.

Participants completed 240 trials across 20 learning runs. Each trial began with a centrally presented fixation cross (jittered between 1–2 s), followed by a facial image displayed above four name options, only one of which was correct (Figure 1). Participants were instructed to select the correct name for the image and respond “as quickly and as accurately as possible,” although a maximum response window of 20 s was allowed. Responses were made via keyboard keys 1–4, with feedback provided via auditory tones (100 ms): a high-pitched tone for correct responses and a low-pitched tone for incorrect ones. The volume of the feedback tones was typically set to 85 dBSPL (decibels sound pressure level). However, volume was adjusted during practice trials to accommodate individual differences in hearing, particularly among older adults.

Following the learning phase and completion of objective interoception measures, participants completed two memory tasks in a fixed order: an associative recall task, followed by an associative recognition task.

**Figure 2:**
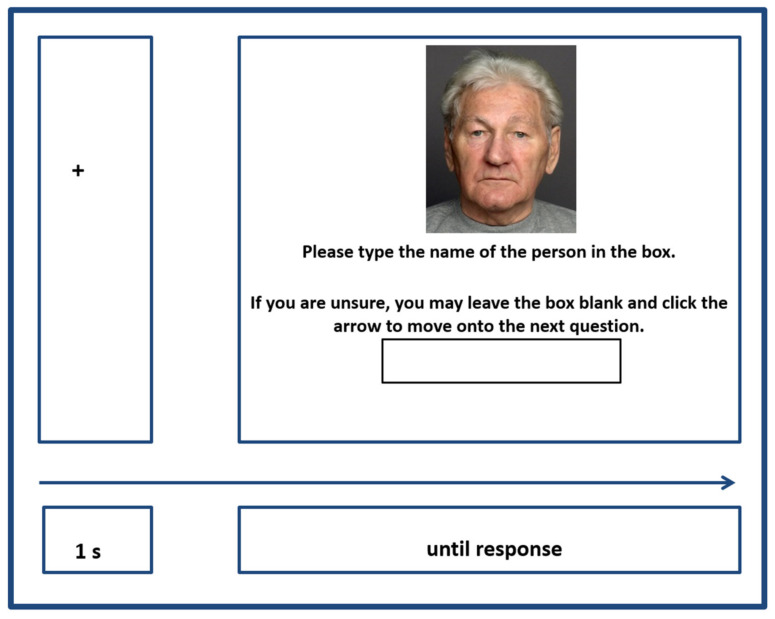
Example of an Associative Cued Recall Trial.

Associative Recall: Participants were presented with each of the twelve studied faces individually and asked to type the corresponding name into a response box (Figure 2). Each face was shown once across 12 trials. The task was self-paced, allowing participants to skip a trial if unsure; however, skipped trials could not be revisited.

**Figure 3:**
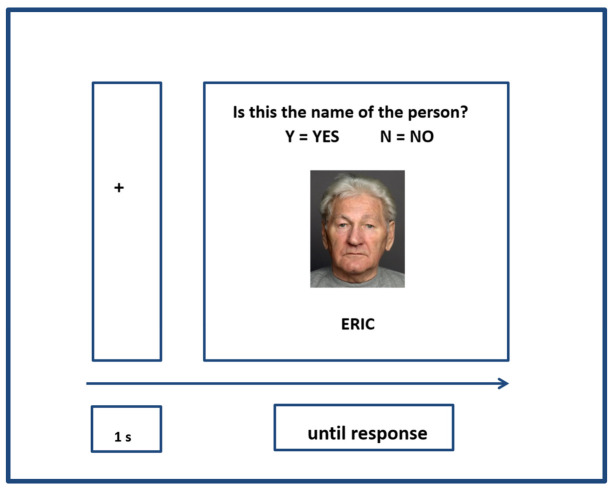
Example of an Associative Recognition Trial.

Associative Recognition: Participants viewed face–name pairs one at a time and indicated whether the pairing was correct using keypress responses (Z = labelled as Y for Yes, N = labelled as N for No). They were instructed to respond “as quickly and as accurately as possible”, and were encouraged to provide their best estimate if unsure, although a maximum response window of 20 s was allowed. Each face was presented 12 times and presented with each possible matching name twice (as split by gender), resulting in a total of 144 randomised trials (see Figure 3).

### 2.5. Equipment

Laboratory testing was conducted using a 24-inch HP monitor positioned approximately 50 cm from the participant. For home-based testing, a 14-inch HP laptop was used at a viewing distance of approximately 40 cm. In both settings, screen resolution was set to 1680 × 1050 to ensure full-screen stimulus presentation. Experimental tasks were programmed using Cogent2000 v1.32 running in MATLAB R2023a (The MathWorks, Inc., Natick, MA, USA). Physiological data were collected using a pulse oximeter to measure heart rate during interoceptive tasks.

Body mass index (BMI) was calculated by measuring participants’ height and weight using standard stadiometers and weighing scales. Participants were asked to remove shoes and empty their pockets during these measurements. BMI values were then computed using the National Heart, Lung, and Blood Institute’s online calculator (https://www.nhlbi.nih.gov/health/educational/lose_wt/BMI/bmicalc.htm (accessed on 29 October 2025)).

### 2.6. Procedure

Participants were initially screened via telephone to confirm eligibility. Screening included questions about neurological, cardiac, and mental health history. Eligible participants were invited to complete a two-part study.

Prior to the in-person session, participants completed demographic questions and two interoceptive questionnaires (BPQ-VSF and MAIA-2) online via Qualtrics. Attention checks were embedded to ensure data quality (e.g., “Please select ‘Almost Always’”). One older adult completed paper versions of the questionnaires, which were returned during the lab session.

In-person testing took place either in a university laboratory or, for a subset of older adults (*N* = 7), in their homes. Home participants were asked to prepare a quiet space with a desk and chair and to allow approximately two hours for uninterrupted testing. To minimise elevated heart rate effects on IAcc (Knapp-Kline & Kline, 2005; Murphy et al., 2018), all participants were instructed to refrain from caffeine for two hours prior to testing.

The session began with BMI measurement, followed by the face–name learning task. Participants then completed the HCT and HDT, each preceded by a practice trial. Confidence ratings were provided after each trial, using a VAS to assess II. The session concluded with the associative recall and recognition memory tasks. Total testing time ranged from 1.5 to 2 h. Participants were reimbursed for their time. Young adults received either SONA points plus a £5 Amazon voucher or a £10 Amazon voucher, while older adults received a £25 Amazon or Love2Shop voucher to reflect their greater time and effort.

## 3. Data Analyses

### 3.1. Preliminary Data Screening and Outliers

All data were checked for parametric assumptions, including normality (Kolmogorov–Smirnov test), homogeneity of variance (Levene’s test), and outliers (Winsorizing technique). Raw questionnaire data met the minimum 90% completion criterion across participants. Accuracy was computed for learning, recall, and recognition tasks, and reaction times for learning and recognition tasks only.

Of the 86 participants, six were excluded as extreme outliers on interoceptive and cognitive measures (z ≥ ±3.29; Field, 2018), resulting in a final sample of 80 (40 younger adults, 40 older adults). No outliers were identified for emotional associative learning or memory tasks when averaging across trials per participant. During the face–name learning paradigm, one younger and eight older participants failed to respond within the 20-s limit at least once but maintained ≥95% task completion and were retained. Missing responses were coded as −999.

One additional outlier was identified for BMI but was not excluded, as BMI was statistically controlled in all interoception-related analyses. A significant group difference in BMI emerged, t(78) = −2.33, *p* = 0.011, d = 3.91, with younger adults (M = 24.16, SD = 3.25) scoring lower than older adults (M = 26.19, SD = 4.47). BMI was included as a covariate, consistent with evidence linking higher BMI to reduced interoceptive sensitivity (Knapp-Kline & Kline, 2005; Willem et al., 2019).

### 3.2. Statistical Analyses

All analyses were conducted using IBM SPSS Statistics (Version 28). Alpha was set at 0.05 (two-tailed) unless otherwise specified. Descriptive statistics (means, standard deviations, sample sizes) and effect sizes (partial η^2^ for ANOVAs, Cohen’s *d* for *t*-tests) are reported throughout.

### 3.3. Interoceptive Sensitivity

Analyses of interoceptive variables addressed hypotheses regarding age differences in interoceptive accuracy, attention, beliefs, and insight. Consistent with evidence that behavioural, self-reported, and metacognitive facets of interoception are largely dissociable (Calì et al., 2015; Garfinkel et al., 2015, 2016a; Murphy et al., 2019; Pearson & Pfeifer, 2022; Suksasilp & Garfinkel, 2022), each measure was examined independently. One-way ANCOVAs tested age group differences across all interoceptive measures while statistically controlling for BMI. Interoceptive Attention was measured using the BPQ-VSF; Interoceptive Beliefs were assessed with the eight MAIA-2 subscales; Interoceptive Accuracy using the HCT and HDT; and Interoceptive Insight was indexed by the correspondence between confidence ratings and behavioural performance following each HCT and HDT trial. This confidence–accuracy correspondence was quantified separately for the HCT and HDT.

For the HCT, Pearson correlations were calculated between six trial-level interoceptive accuracy scores and the corresponding confidence ratings for each participant, yielding an r-value that indicated the relationship strength between participants’ confidence ratings and their actual performance. A measure of HCT II could not be ascertained for one older participant due to consistently reporting confidence at 100%. Therefore, for all HCT II-specific analyses, data are reported for 39 older adults.

For the HDT, Receiver Operating Characteristic (ROC) curve analysis was applied, following Garfinkel et al. (2015), to evaluate how well confidence ratings predicted accuracy. This approach incorporated hit rates (accurate trials endorsed with high confidence) and false alarm rates (inaccurate trials endorsed with high confidence), providing a measure of metacognitive awareness of interoceptive performance.

### 3.4. Emotional Associative Learning

Learning trajectories for emotional face–name associations were modelled separately for accuracy and response time. These analyses addressed hypotheses regarding age and emotion effects on learning, including the expected positivity-related advantage in older adults. Learning Accuracy was expressed as the proportion of correct responses, and these values were averaged across participants for each run (2–20) and each emotional valence (angry, neutral, happy). Run 1 was excluded because it served as the initial familiarisation phase: participants had not yet received feedback and could only guess the face–name pairings. Including this trial-and-error exposure round would therefore have conflated novelty-driven responding with genuine learning.

A logarithmic function was fitted to the proportion of correct responses using the equation: y = A + B⋅ln(x) to estimate the rate of change in accuracy across task runs. Logarithmic functions are appropriate for assessing performance improvement across trials, reflecting rapid early learning before reaching a plateau (Heathcote et al., 2000).

Response time data for correct trials were modelled using an exponential function, y = A⋅e^B⋅x^, to capture the rate of change in response speed over time, in line with prior research assessing changes in performance accuracy and speed over multiple trials (Vianna et al., 2024; Zhang et al., 2025).

Slope estimates derived from each curve were entered into SPSS and analysed using 2 (age group: younger, older) × 3 (emotion: angry, neutral, happy) ANOVAs to assess age-related differences in learning rate for both accuracy and response time.

### 3.5. Interoception–Emotional Associative Learning Relationships

The interoception–learning analyses focused on whether interoceptive measures showed stronger relationships with emotional associative learning in younger adults, reflecting theoretical accounts that ageing weakens the links between bodily states and emotional experience (Mendes, 2010; MacCormack et al., 2022). One-tailed partial correlations (controlling for BMI) were computed between interoceptive measures and emotional associative learning accuracy (averaged across runs 18–20 where accuracy was highest). Correlations were computed separately for each age group, emotion condition, and interoceptive measure.

To control for the familywise error rate across multiple comparisons, the Larzelere and Mulaik (1977) correction was applied. Fisher’s Z transformations were used to assess whether the strength of the correlations significantly differed between age groups.

### 3.6. Emotional Associative Memory

Analyses of emotional associative memory examined whether memory performance differed as a function of age and emotional valence, with expectations that older adults would show advantages for happy face–name pairs and that younger adults might show reduced performance for angry pairs due to attentional narrowing toward negative emotion. Three mixed-measures ANOVAs with a 2 × 3 design (age group × emotion) examined cued recall accuracy, recognition accuracy, and response times for correctly recognised trials.

Age (younger, older adults) was treated as a between-subjects factor, and emotional valence (angry, neutral, happy) as a within-subjects factor. Bonferroni corrections were applied to adjust the significance threshold for all post hoc pairwise comparisons, thereby controlling the familywise Type I error rate across multiple comparisons between emotional valence conditions.

### 3.7. Interoception–Emotional Associative Memory Relationships

These analyses explored whether the relationship between interoception and emotional associative memory differed across age groups, with associations expected to be stronger in young adults (Mendes, 2010; MacCormack et al., 2022). Partial correlations (controlling for BMI) were computed separately for each age group, emotion, and interoceptive measure (HCT, HDT with II scores, BPQ-VSF, and MAIA-2 subscales). The Larzelere and Mulaik (1977) correction was again applied to account for multiple comparisons. Fisher’s Z transformations were used to test for significant differences in correlation strength between younger and older adults.

All statistical assumptions were checked and met, including homogeneity of variance, sphericity, and linearity. Significance thresholds for Fisher’s Z transformations were denoted as † *p* < 0.05 and ††† *p* < 0.001.

## 4. Results

### 4.1. Age Differences in Interoception

**Figure 4:**
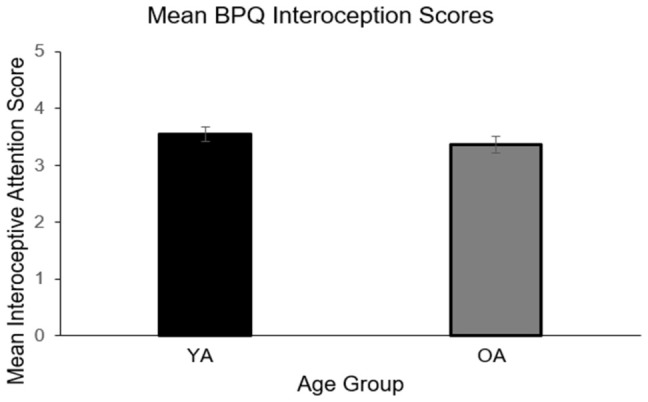
Age Differences in Interoceptive Attention. Note. This figure represents experimental findings for mean interoceptive attention (measured by the BPQ-VSF; The Very Short Form Body Perception Questionnaire. YA = Young adults, OA = Older adults. Planned contrasts for between-subject comparisons with each bar representing mean interoceptive attention scores ± SEM.

Interoceptive attention, measured by the BPQ-VSF, did not significantly differ between young and older adults (F(1, 77) = 1.68, *p* = 0.199; ηp^2^ = 0.021), as shown in Figure 4.

**Figure 5:**
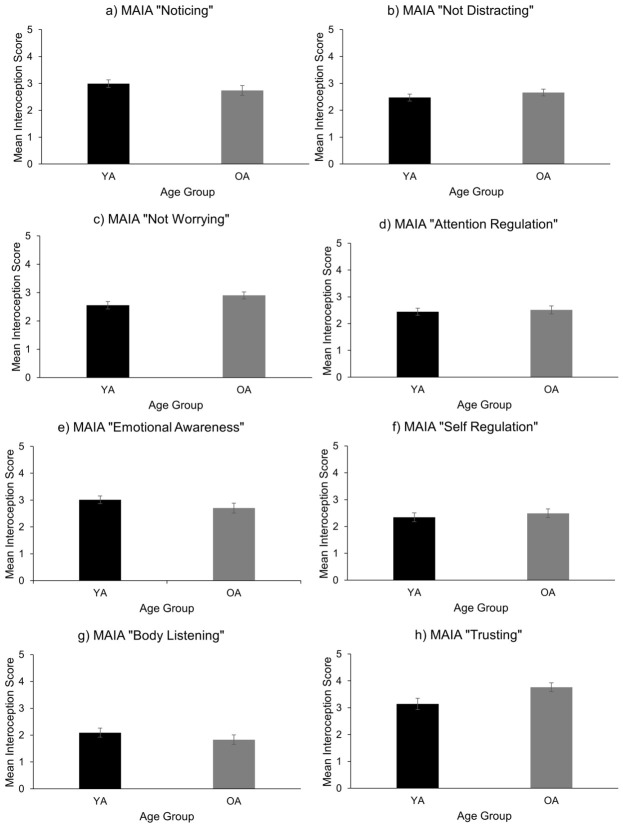
Age Differences in Interoceptive Beliefs. Note. This figure represents experimental findings for self-reported interoceptive belief scores when measured by the MAIA-2 as a multidimensional construct concerning (**a**) noticing, (**b**) not distracting, (**c**) not worrying, (**d**) attention regulation, (**e**) emotional awareness, (**f**) self-regulation, (**g**) body listening and (**h**) trusting, across age groups. MAIA = Multidimensional Assessment of Interoceptive Awareness. YA = Young adults, OA = Older adults. * *p* < 0.05, sig. different from young adults. Planned contrasts for between-subject comparisons with each bar representing mean interoceptive belief scores ± SEM.

By contrast, significant age differences emerged in interoceptive beliefs, measured using the MAIA-2. Older adults reported marginally significantly less worry about their bodily sensations (F(1, 77) = 4.28, *p* = 0.042, ηp^2^ = 0.053) and significantly greater trust in their internal signals (F(1, 77) = 5.20, *p* = 0.025, ηp^2^ = 0.063) compared to younger adults (see Figure 5c,h). No significant age differences were found across the remaining MAIA-2 subscales. Notably, BMI was negatively correlated with the Noticing subscale (r = −0.311, *p* = 0.003, ηp^2^ = 0.082), suggesting that higher BMI was associated with reduced awareness of bodily signals.

Interoceptive accuracy, assessed via the HCT and HDT, did not differ significantly between younger and older adults, F(1, 77) = 0.59, *p* = 0.447, ηp^2^ = 0.008 and F(1, 77) = 2.92, *p* = 0.092, ηp^2^ = 0.036, respectively. Similarly, interoceptive insight, derived from confidence ratings on both tasks, showed no significant age differences (F < 0.051, *p* > 0.822, ηp^2^ < 0.001).

### 4.2. Age Differences in Emotional Associative Learning

#### 4.2.1. Learning Rate

Learning trajectories were modelled using logarithmic curve fitting. No main effect of age was found (F(1, 36) = 3.15, *p* = 0.084, ηp^2^ = 0.081), but there was a significant main effect of emotion (F(1.98, 71.43) = 24.99, *p* < 0.001, ηp^2^ = 0.410). Results further highlighted a significant age × emotion interaction (F(1.97, 71.43) = 2.22, *p* < 0.001, ηp^2^ = 0.438). Younger adults learned angry face–name pairs faster than older adults (*p* = 0.019), with no further group differences for learning neutral and happy face–name pairs (all *p* > 0.102).

**Figure 6:**
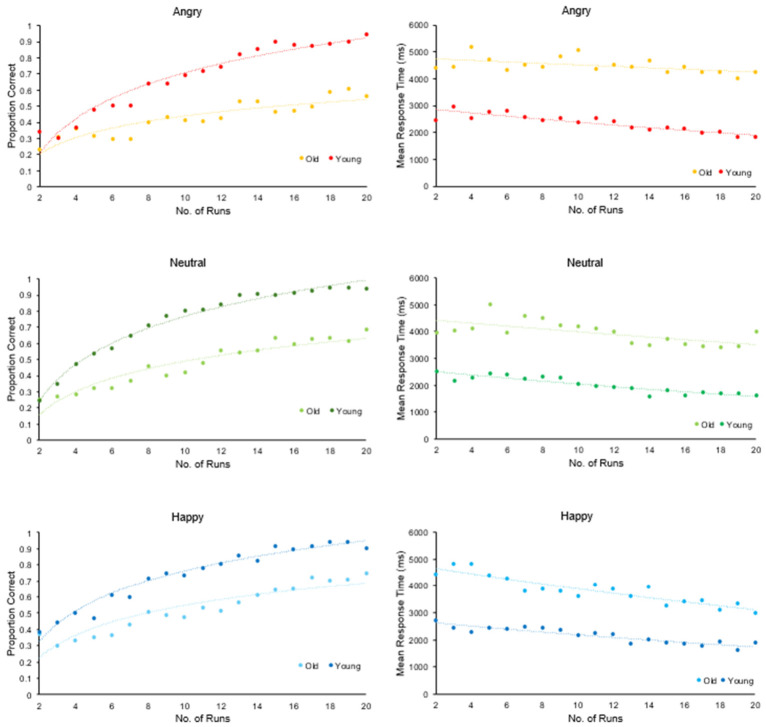
Learning Curves for Accuracy and Response Time in the Emotional Associative Face–name Learning Paradigm. Note. The y-axes present mean proportion of correct responses and mean response time (RT) for correct responses for each emotion. The x-axes represent learning runs across the task. The second run marks the point after one full trial-and-error learning cycle has occurred.

Within-group analysis revealed that young adults learned happy face–name pairs significantly slower than angry and neutral ones (all *p* < 0.001), with no difference between angry and neutral trials (*p* = 0.069). In contrast, older adults showed faster learning for happy and neutral pairs compared to angry ones (all *p* < 0.001), with no difference between happy and neutral trials (*p* > 0.999) (see Figure 6).

#### 4.2.2. Response Times Across Learning Progression

Exponential curve modelling of response times showed no significant effect of age (F(1, 36) = 2.12, *p* = 0.154, ηp^2^ = 0.056) but a significant main effect of emotion (F(1.52, 54.88) = 267.35, *p* < 0.001, ηp^2^ = 0.881) and a significant age × emotion interaction (F(1.52, 54.88) = 364.98, *p* < 0.001, ηp^2^ = 0.910).

Pairwise comparisons revealed that older adults showed greater improvement in response times for happy face–name pairs than younger adults (*p* < 0.001), but less improvement for angry pairs (*p* < 0.001). No age difference was found for neutral pairs (*p* = 0.611).

Within-group analysis revealed that younger adults were slower to respond to angry face–name pairs compared to neutral (*p* = 0.005), with no other differences identified (*p* > 0.059). By contrast, older adults responded faster to happy face–name pairs and slower to angry ones, with neutral responses in between (all differences *p* < 0.001) (see Figure 6).

### 4.3. Interoception–Emotion Relationships: Learning

To explore the relationship between interoception and emotional associative learning, partial correlations (controlling for BMI) were computed separately for each age group and emotional valence. Among younger adults, greater interoceptive attention (BPQ-VSF) was significantly positively associated with learning neutral face–name pairs (r = 0.469 *), while greater interoceptive insight (HDT II) was significantly negatively associated with neutral pair learning (r = −0.441 *). In older adults, greater interoceptive accuracy (HCT) was significantly positively associated with learning angry face–name pairs (r = 0.356 *).

Fisher’s Z transformations revealed significant age differences in the strength and direction of these relationships. Specifically, the relationship between the MAIA-2 Noticing subscale and learning happy face–name pairs differed significantly between groups (Z = −2.24, *p* = 0.013), as did the relationship between MAIA-2 Not Worrying and learning neutral face–name pairs (Z = 1.87, *p* = 0.031). No other significant age differences were found for the association between interoception and emotional learning accuracy. Refer to Table S1 in the Supplementary Materials for full comparison data.

### 4.4. Age-Related Differences in Emotional Associative Memory

#### 4.4.1. Accuracy

Emotional associative memory accuracy was assessed through cued recall and recognition tasks. Analysis of cued recall yielded a significant main effect of age (F(1, 78) = 61.22, *p* < 0.001, ηp^2^ = 0.440), and a significant main effect of emotion (F(1.93, 150.12) = 5.14, *p* = 0.007, ηp^2^ = 0.062). This was qualified by an age × cued recall interaction (F(1.93, 150.12) = 7.09, *p* = 0.001, ηp^2^ = 0.083). Associative recognition showed a significant main effect of age (F(1, 78) = 61.50, *p* < 0.001, ηp^2^ = 0.441), and a significant main effect of emotion (F(1.90, 148.34) = 7.66, *p* < 0.001, ηp^2^ = 0.089). This was qualified by a significant age × associative recognition interaction (F(1.90, 148.63) = 4.14, *p* = 0.019, ηp^2^ = 0.050). Pairwise comparisons showed that in both recall and recognition tasks, younger adults outperformed older adults across all emotional categories (all *p* values < 0.001).

**Figure 7:**
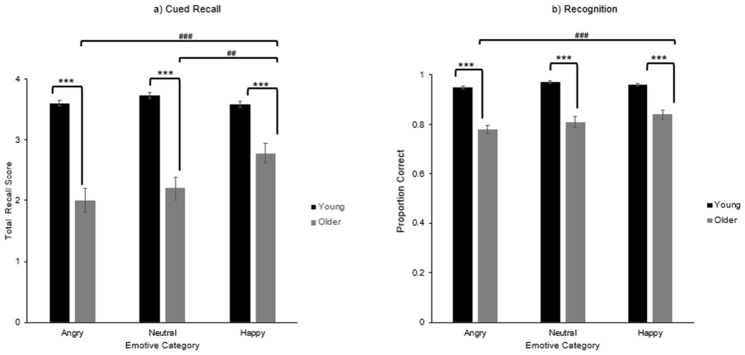
Age Differences in Emotional Associative Memory Accuracy in Cued Recall and Recognition. Note. This figure represents age differences in emotional associative memory: (**a**) correct cued recall and (**b**) proportion of correct recognition. Age × Emotion interaction: *** *p* < 0.001, sig. different between age groups, ^##^ *p* < 0.01, ^###^ *p* < 0.001, sig. different between emotional associative memory in older adults. Bonferroni post hoc comparison for between-subject comparisons with each bar representing mean emotional associative learning accuracy scores ± SEM.

Within-group analyses for cued recall showed that older adults recalled happy face–name pairs significantly more accurately than angry (*p* < 0.001) and neutral (*p* = 0.002) pairs, with no difference between angry and neutral pairs (*p* = 0.822). No emotional valence-related differences in recall were observed among younger adults (*p* > 0.999) (see Figure 7a).

Within-group analyses for recognition showed that older adults recognised happy face–name pairs significantly more accurately than angry ones (*p* < 0.001), with no other significant differences observed (*p* > 0.065). In contrast, younger adults’ recognition accuracy did not vary significantly across emotions (*p* > 0.208) (see Figure 7b).

#### 4.4.2. Response Times for Recognition

For accurate recognition trials, young adults responded significantly faster than older adults, F(1, 78) = 80.11, *p* < 0.001, ηp^2^ = 0.507. There was also a significant main effect of emotion on response time, F(1.84, 143.65) = 15.38, *p* < 0.001, ηp^2^ = 0.165), and a significant age × emotion interaction, F(1.84, 143.65) = 3.99, *p* = 0.024, ηp^2^ = 0.049.

**Figure 8:**
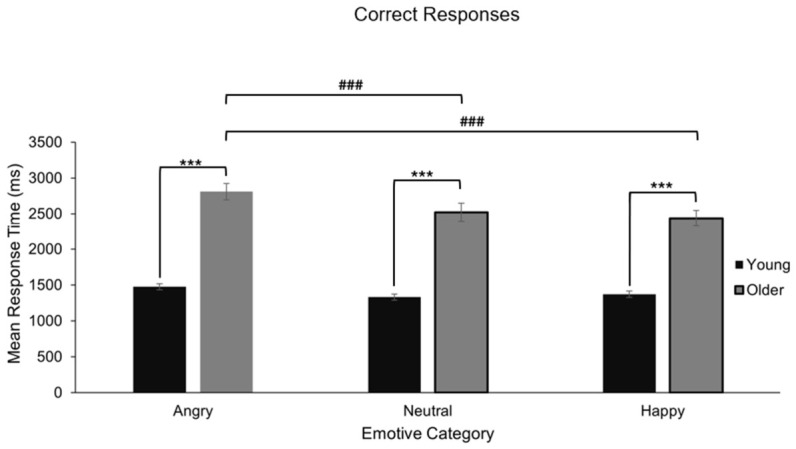
Response Latencies for Correct Emotional Associative Recognition Across Age Groups. Note. This figure depicts mean differences in response time between young and older adults for correct responses in emotional associative recognition.Age × Emotion interaction: *** *p* < 0.001, sig. different between age groups, ^###^ *p* < 0.001, sig. different between emotive category in older adults. Bonferroni post hoc comparison for between-subjects comparisons with each bar representing mean emotional associative recognition accuracy scores ± SEM.

Pairwise comparisons revealed that older adults were significantly slower in recognising angry face–name pairings compared to happy and neutral ones (*p* < 0.001), which did not differ from each other (*p* = 0.445). Young adults showed no significant differences in response time across emotions (*p* > 0.143), with performance remaining stable (Figure 8).

### 4.5. Interoception–Emotion Relationships: Memory

#### 4.5.1. Cued Recall

In both young and older adults, three moderate but statistically significant associations were found between behavioural measures of interoception and accurate recall of angry face–name pairings. Among young adults, greater recall of angry face–name pairs was significantly associated with lower IAcc (HCT: r = −0.359, *p* < 0.05) and lower interoceptive insight (HCT II: r = −0.342, *p* < 0.05). Similarly, older adults showed a significant negative correlation between IAcc (HDT: r = −0.364, *p* < 0.05) and recall. These findings suggest that, across age groups, higher interoceptive sensitivity may be linked to poorer memory for information paired with angry expressions.

However, Fisher’s Z transformations revealed significant age differences in the strength and direction of associations between interoceptive measures and recall of angry face–name pairings. While young adults showed a positive relationship between interoceptive attention (BPQ) and recall of angry face–name pairs, older adults showed a negative association—this contrast was statistically significant (Z = 2.67, *p* < 0.001). Conversely, for interoceptive accuracy (HCT) and interoceptive insight (HCT II), young adults showed negative associations with recall of angry face–name pairs, while older adults showed positive ones. These age-related differences were statistically significant (HCT: Z = −1.78, *p* = 0.034; HCT II: Z = −2.10, *p* = 0.018), suggesting that in older adulthood, more accurate and confident perception of bodily signals may support better recall memory for information presented in the context of anger.

Details on age group differences, emotional associative recall accuracy, interoception, and correlation strengths are provided in the Supplementary Materials (Table S2).

#### 4.5.2. Recognition

Only one significant correlation was observed between interoception and associative emotion recognition: older adults showed a moderate negative relationship between IAcc (HDT) and recognition of angry face–name pairs (r = −0.407, *p* < 0.05).

Fisher’s Z transformations revealed significant age differences in the direction and strength of associations between interoception and associative emotion recognition:

Interoceptive attention (BPQ-VSF) and recognition of neutral face–name pairs differed significantly between age groups (Z = 2.34, *p* = 0.010), with a weak positive correlation in young adults (r = 0.261) contrasting a weak negative correlation in older adults (r = −0.269).

Interoceptive beliefs (MAIA-2) showed age-related directional differences across several subscales. For the Noticing subscale, a significant difference emerged for the recognition of neutral face–name pairs (Z = 1.70, *p* = 0.045). Attention Regulation also showed a significant difference for the recognition of neutral face–name pairs (Z = 2.35, *p* = 0.009). Finally, Body Listening revealed significant differences for the recognition of both neutral (Z = 2.06, *p* = 0.020) and angry face–name pairs (Z = 2.06, *p* = 0.043). Across these relationships, younger adults tended to show weak positive correlations, while older adults showed weak to moderate negative correlations.

Interoceptive accuracy (HDT) and recognition of angry face–name pairs differed significantly between age groups (Z = 2.69, *p* = 0.004), with a non-significant positive correlation in young adults (r = 0.191) and a significant negative correlation in older adults (r = −0.407).

Interoceptive insight (HCT II) showed significant age differences in the recognition of emotional face–name pairs. Specifically, older adults differed from younger adults in recognising neutral (Z = −1.97, *p* = 0.024) and happy face–name pairs (Z = −2.03, *p* = 0.021). Notably, HCT II was the only interoceptive measure where older adults showed positive correlations with associative emotion recognition, while younger adults showed negative correlations—reversing the age-related pattern observed across other interoceptive domains.

Full details of age group differences, emotional recognition accuracy, interoception, and correlation strength comparisons are available in the Supplementary Materials (Table S3).

## 5. Discussion

This study explored how interoceptive processes relate to emotional learning and memory across the adult lifespan, integrating biological and cognitive frameworks to understand age-related changes. The findings offer insights into how the mind–body connection evolves with age, supporting the concept of maturational dualism (Mendes, 2010) and extending the Socioemotional Selectivity Theory (Carstensen, 1993, 2006) by linking emotional preferences with interoceptive sensitivity.

### 5.1. Interoceptive Sensitivity Across Age

Contrary to our hypotheses, we found no significant age differences in interoceptive accuracy (HCT, HDT), interoceptive attention (BPQ-VSF), or interoceptive insight (confidence ratings). While most studies report an age-related decline in behavioural interoceptive accuracy (Lohani, 2014; Murphy et al., 2018, 2019; Nusser et al., 2020; Khalsa et al., 2009b; Dobrushina et al., 2024), often attributed to sensory and physiological changes (Völter et al., 2021; Boss & Seegmiller, 1981) that weaken the integration of bodily sensations and emotional experience (MacCormack et al., 2022; Mendes, 2010), a smaller body of research suggests stable IAcc across age groups (Mikkelsen et al., 2019; Dobrushina et al., 2020) or even enhanced interoceptive insight in later life (Nusser et al., 2020). Our findings add to this emerging evidence, suggesting that the ability to detect and reflect on bodily signals may remain intact for some older adults. This interpretation is reinforced by the absence of age differences in interoceptive attention (BPQ-VSF), despite previous reports of decline in this dimension (Murphy et al., 2018; Ulus & Aisenberg-Shafran, 2022; Elliot & Pfeifer, 2022). One possible explanation is the well-documented increase in variability across performance measures in older adults (Rutter et al., 2020; Correia et al., 2018), including interoceptive tasks. For instance, Haustein et al. (2023) reported substantial individual differences in IAcc within an older sample aged 60–91 years, with some participants achieving up to 97% accuracy on the HCT. Similarly, Cioffi et al. (2017) found that older adults outperformed younger adults on the HCT. These findings suggest that age-related effects may be obscured by heterogeneity in physiological and cognitive ageing trajectories.

Another possible explanation for the absence of an age-related decline in IAcc in our study is the limited control over several confounding variables known to influence IAcc across the lifespan. Factors such as time perception, cardiac function, BMI, and heart rate beliefs can influence interoceptive performance, and when statistically controlled, age-related differences in IAcc may diminish or disappear (Murphy et al., 2019; Haustein et al., 2023). However, findings across studies remain inconsistent: Some report persistent age-related declines even after accounting for these confounds (Murphy et al., 2018), while others find no decline regardless of controls (Ueno et al., 2020). The lack of comprehensive control in our study therefore represents a methodological limitation and may have contributed to the null findings regarding age-related differences, specifically in IAcc.

Nonetheless, a strength of our study is the inclusion of BMI as a covariate, particularly given that older adults in our sample exhibited significantly higher BMI than younger adults. Elevated BMI has been associated with reduced interoceptive sensitivity across multiple dimensions (Willem et al., 2019; Robinson et al., 2021). Specifically, our results showed that BMI was negatively correlated with scores on the MAIA-2 Noticing subscale. This pattern converges with Willem et al. (2019), who reported significant reductions in body awareness on the Noticing and Trusting subscales of the MAIA, demonstrating that BMI is associated with multiple interoceptive dimensions, including self-report measures.

Significant age differences emerged in interoceptive beliefs: older adults reported greater trust and less worry about bodily sensations, consistent with prior findings that suggest selective changes in belief-based interoception with age (Elliot & Pfeifer, 2022; Ross & Gillett, 2019). These results support the notion that interoceptive beliefs may be shaped by accumulated bodily experience and emotional regulation strategies in later life.

### 5.2. Emotional Associative Learning and Memory

As predicted, older adults demonstrated a positivity bias, learning and recalling happy face–name pairs more accurately and faster than angry or neutral ones. This pattern was evident across learning accuracy, response times, and memory tasks, and is consistent with Socioemotional Selectivity Theory (Carstensen, 1993, 2006), which proposes that older adults prioritise emotionally meaningful and positive information due to perceived time limitations. By contrast, younger adults showed emotionally neutral performance, with consistent accuracy and speed across emotional categories.

The attentional narrowing hypothesis (Easterbrook, 1959) posits that high-arousal emotions focus attention on central emotional stimuli, reducing processing of peripheral details such as names. This mechanism was expected to impair associative memory for information paired with negative stimuli, particularly in younger adults (Rimmele et al., 2011). However, learning rate analyses revealed that younger adults responded faster than older adults across all emotional conditions, and their overall performance remained stable. Although within-group comparisons indicated slower learning for angry face–name pairs relative to neutral, this did not translate into a clear associative memory deficit. One possibility is that the emotional facial expressions used did not elicit sufficient arousal to trigger attentional narrowing. Additionally, younger adults’ broader attentional capacity may have buffered against emotion-induced narrowing, resulting in relatively consistent performance across emotional categories.

By contrast, older adults demonstrated a clear positivity bias, learning and remembering happy face–name pairs more accurately and faster than angry or neutral ones. This advantage was evident not only during learning but also in recall and recognition tasks, highlighting a consistent preference for positive emotional content in later life. Importantly, these findings allow us to rule out attentional narrowing as a mechanism in older adults: if attentional narrowing had occurred, it would have impaired memory for information paired with happy stimuli, which was not observed. Instead, older adults showed enhanced performance for happy face–name pairs, suggesting that age-related changes in visceral arousal may promote reliance on cognitive appraisal processes (Barrett, 2017; MacCormack et al., 2021), thereby favouring emotionally positive associations.

The combination of slower, yet less accurate responses to angry face–name pairs in older adults are unlikely to reflect attentional narrowing, which should have enhanced associative learning and memory for emotionally salient stimuli. Instead, this pattern suggests reduced engagement with negative stimuli, consistent with motivational shifts toward positivity and emotional wellbeing in ageing (Bjalkebring et al., 2015; English & Carstensen, 2014; Scheibe et al., 2013; Hay & Diehl, 2011). Age-related changes in emotional reactivity and arousal, possibly driven by sensory decline (Völter et al., 2021) and physiological changes (Boss & Seegmiller, 1981), may have contributed to this reduced responsiveness to negative stimuli, reinforcing a selective preference for positive emotional content.

### 5.3. Interoception–Emotion Relationships

This section addresses the final research question by examining whether interoceptive processes differentially relate to emotional associative learning and memory across adulthood. As anticipated, interoception–emotion relationships were stronger and more consistent in younger adults, consistent with tighter mind–body coupling earlier in adulthood (Lohani, 2014; Mikkelsen et al., 2019; Ulus & Aisenberg-Shafran, 2022). In contrast, older adults showed more differentiated and valence-specific associations, indicating age-related shifts in how bodily signals contribute to emotional cognition. Importantly, these effects varied across interoceptive dimensions and memory stages, highlighting the need to distinguish encoding from retrieval processes when interpreting interoception–emotion interactions across the lifespan.

### 5.4. Interoception–Emotion Relationships: Emotional Associative Learning

At the level of encoding, clear age-related differences emerged in how interoceptive processes influenced emotional associative learning. Although interoceptive attention and beliefs did not differ between age groups at the level of mean performance on the BPQ-VSF and MAIA-2 Noticing subscale, they related differently to learning outcomes. For MAIA-2 Noticing, a significant age difference emerged for happy face–name pairs: higher Noticing was associated with slower learning in older adults but faster learning in younger adults. This does not indicate poorer overall learning in older adults; rather, within this group, greater bodily noticing reduced the magnitude of the positivity bias, which was strongest among those with lower interoceptive noticing. These findings suggest that the positivity bias, typically framed as a motivational and cognitive shift within Socioemotional Selectivity Theory (SST; Carstensen, 1993, 2006), also has a physiological component. Specifically, interoceptive beliefs might modulate the prioritisation of positive information at encoding. Consistent with the physiological hypothesis of emotional ageing (PHEA; MacCormack et al., 2022), reduced reliance on bodily signals in later life may support greater top-down regulation and appraisal-based processing (Barrett, 2017; MacCormack et al., 2021), thereby strengthening positivity-driven learning. To our knowledge, this provides novel evidence that interoceptive beliefs contribute to age-related variability in the strength of the positivity bias during associative learning.

This pattern extended to the MAIA-2 Not Worrying subscale. Among younger adults, greater bodily worry was associated with better associative learning of neutral face–name pairs, suggesting that heightened bodily arousal may facilitate encoding of low-arousal or emotionally ambiguous information. This interpretation aligns with evidence that visceral signals can enhance memory for neutral stimuli by strengthening visceral–cognitive coupling (Fiacconi et al., 2016; Garfinkel et al., 2013) and is consistent with maturational dualism, which proposes tighter integration between bodily states and cognition in young adulthood (Mendes, 2010). By contrast, older adults showed only a weak positive association between Not Worrying and neutral learning, indicating minimal coupling between bodily worry and associative encoding. The significant difference in both strength and direction of these associations across age groups points to an age-related shift in mind–body coupling and its functional role in emotional associative learning. This decoupling is consistent with age-related reductions in physiological responsiveness (Boss & Seegmiller, 1981) and decreased signal-to-noise in interoceptive afferents, as described by the Physiological Hypothesis of Emotional Ageing (MacCormack et al., 2022).

Behavioural IAcc revealed a more selective and theoretically informative pattern that complements the broader age-related decoupling described above. Older adults with higher IAcc, measured using the heartbeat counting task (HCT), showed better associative learning of faces expressing high-arousal emotions such as anger. This pattern points to a compensatory mechanism at the encoding stage, whereby preserved sensitivity to visceral signals partially offsets age-related declines in physiological reactivity and interoceptive precision. According to the physiological hypothesis of emotional ageing (PHEA; MacCormack et al., 2022), ageing weakens the coupling between bodily signals and emotional experience, shifting emotional processing away from sensory-driven mechanisms toward greater reliance on cognitive appraisal (MacCormack et al., 2021; Barrett, 2017). Within this framework, heightened sensitivity to cardiac signals in a subset of older adults may temporarily restore the salience of highly arousing stimuli, such as angry faces, thereby supporting associative encoding despite broader physiological decline (Boss & Seegmiller, 1981).

Notably, older adults overall still learned happy face–name pairs more accurately than angry ones, and this positivity advantage was unrelated to IAcc. Thus, IAcc modulated the encoding of angry information without overriding the dominant positivity bias. Attentional narrowing can also be ruled out at the learning stage: older adults with better IAcc showed enhanced, rather than impaired, encoding of angry associations, contrary to threat-driven narrowing accounts (Rimmele et al., 2011). Taken together, the positive association between HCT accuracy and angry learning is best interpreted as a targeted compensatory mechanism that enables efficient binding of emotionally salient negative information in older adults who otherwise prioritise positive content.

### 5.5. Interoception–Emotion Relationships: Emotional Associative Recall

The relationship between interoception and recall memory revealed a pronounced age-related divergence, particularly for anger. Recall relies primarily on recollection-based processes (Naveh-Benjamin et al., 2009; Grady et al., 2003), making it especially sensitive to the effects of heightened emotional arousal and attentional narrowing.

Among younger adults, greater IAcc (HCT) and insight (HCT II) were associated with poorer recall of names associated with angry faces. This aligns with classical emotional arousal theories (James, 1884; Lange, 1885), which propose that heightened bodily awareness intensifies emotional responses. Supporting this interpretation, threat perception studies (Pfeifer et al., 2017; Garfinkel & Critchley, 2016) have shown that interoceptive signals enhance attention to emotionally salient stimuli. In this context, attentional narrowing (Easterbrook, 1959) may explain why, during recall, when faces were presented and the recall of names was required, young adults with high IAcc were more attentive to angry faces, interfering with recall processes, and thus reducing name recall.

In older adults, a related but more differentiated pattern emerged. Greater interoceptive attention, indexed by the BPQ-VSF, was associated with poorer recall of angry face–name pairs, suggesting that heightened attention to bodily signals during retrieval amplified threat salience and disrupted associative recall. A similar pattern was observed for behavioural IAcc: older adults with higher IAcc measured using the HDT showed impaired recall of angry associations. As the HDT requires integration of internal cardiac signals with external auditory cues, stronger performance may reflect preserved interoceptive integration. In emotionally salient contexts, such preserved integration may increase sensitivity to angry stimuli, leading to attentional narrowing and reduced recollection of associated names.

Notably, this recall pattern diverged from learning in older adults. While higher IAcc measured with the HCT was positively associated with encoding of angry face–name pairs, recall and recognition showed negative associations with IAcc, particularly when assessed using the HDT (Garfinkel et al., 2015). This dissociation suggests that while bodily attention and preserved visceral sensitivity may support the initial encoding of emotionally salient material, interoceptive integration during retrieval amplifies threat sensitivity, leading to attentional narrowing and impaired associative recall. Together, these findings indicate that the functional role of interoception shifts across memory stages and with age: bodily signals may facilitate encoding under certain conditions but become disruptive during recollective retrieval of high-arousal negative information.

### 5.6. Interoception–Emotion Relationships: Emotional Associative Recognition

Recognition memory, which can be supported by familiarity as well as recollection (Yonelinas et al., 2024; Nie & Wu, 2023), showed both continuity and divergence from recall patterns. Older adults again exhibited a significant negative association between IAcc, measured using the HDT, and recognition of angry face–name pairs, mirroring the recall findings. This convergence across retrieval stages indicates that intact interoceptive integration may heighten arousal responses to anger at retrieval, narrowing attention even when familiarity cues are available, thereby disrupting recognition of associated contextual details.

Age-related differences were particularly pronounced for neutral face–name recognition. Interoceptive attention (BPQ-VSF) and interoceptive beliefs (MAIA-2 Noticing, Attention Regulation, Body Listening) showed significant differences between age groups. Younger adults demonstrated weak positive relationships between these interoceptive dimensions and recognition of neutral face–name associations, whereas older adults showed weak to moderate negative relationships. This pattern suggests that heightened bodily awareness in later life may bias interpretation of ambiguous or neutral facial expressions toward threat (Gray et al., 2007), resulting in attentional narrowing and poorer associative recognition. In contrast, younger adults may continue to recruit bodily signals to support recognition when emotional meaning is ambiguous, consistent with tighter mind–body coupling earlier in adulthood.

Interoceptive insight (HCT II) emerged as the only interoceptive dimension in which older adults showed positive relationships with recognition of neutral and happy face–name pairs, significantly differing from the negative associations observed in younger adults. Although these within-group associations were modest, their consistent reversal across age groups suggests that metacognitive awareness of bodily signals, rather than raw IAcc or subjective beliefs and attention, may uniquely support familiarity-based emotional recognition in later life, particularly for stimuli aligned with motivational goals (Fiacconi et al., 2016; Garfinkel et al., 2013).

## 6. Limitations and Future Directions

Despite these theoretical contributions, several methodological limitations should be acknowledged. First, behavioural IAcc was assessed exclusively using cardiac measures (HCT and HDT). While cardiac interoception remains the most commonly used behavioural index, interoceptive signals arise from multiple bodily systems, including respiratory, urinary, and gastrointestinal domains (Heeringa et al., 2011; Van Oudenhove et al., 2016; Garfinkel et al., 2016a). Restricting assessment to cardiac modalities therefore limits comparability with studies employing non-cardiac measures. This is particularly relevant for emotional processing, given evidence that emotion regulation is closely linked to respiratory signalling (Harrison et al., 2021), and may partially account for the absence of robust age differences in IAcc observed here.

Second, the absence of a time-estimation control task limits interpretation of HCT performance, as it remains unclear whether task success reflected interoceptive sensitivity or age-related differences in temporal perception (Murphy et al., 2018). Time perception is known to change with age and is shaped by attentional, memory, and neural mechanisms that overlap with interoceptive processing (Lustig & Meck, 2011; Turgeon et al., 2016). Moreover, subjective time perception is closely linked to monitoring internal bodily rhythms via insular integration mechanisms (Wittmann, 2009, 2013, 2015). Incorporating time-estimation controls in future research would help disentangle temporal cognition from interoceptive accuracy across adulthood.

Third, emotional processing was inferred from associative learning and memory for emotional facial expressions, rather than from directly experienced or momentary emotional states (Wilhelm & Grossman, 2010). Although this paradigm captures emotionally valenced cognition, it does not assess lived emotional experience under conditions of high arousal. As interoceptive processes are more strongly engaged during emotionally intense and dynamically unfolding experiences (Dunn et al., 2010; Scheibe et al., 2013), this may have constrained observed interoception–emotion associations.

Future research would benefit from integrating ecologically valid methodologies that capture emotion as it unfolds in daily life. In particular, Experience Sampling Methods (ESMs) offer a promising approach to examining fluctuations in interoceptive attention, bodily awareness, and emotional experience across contexts and time (Scollon et al., 2003; Brans et al., 2013; Mikkelsen et al., 2024). Combining ESM with state-level measures of interoceptive attention (Poerio et al., 2024) and physiological monitoring would extend laboratory findings and clarify how interoceptive processes dynamically shape emotional regulation across adulthood.

## 7. Conclusions

This study demonstrated that interoceptive sensitivity remained relatively stable across the adult lifespan, with no significant age differences in interoceptive accuracy, attention, or insight. However, older adults reported more positive interoceptive beliefs, including greater trust in, and reduced worry about, bodily sensations, indicating selective age-related changes in belief-based interoception. Emotional associative learning and memory in older adults were shaped by a consistent positivity bias, with enhanced performance for names paired with happy faces across learning, recall, and recognition tasks. In contrast, younger adults showed more uniform associative emotional processing across happy, angry and neutral valence.

Interoceptive processes were differentially linked to emotional cognition across age groups. Taken together, our findings demonstrate that age-related changes in emotional learning and memory are not purely cognitive but are partly grounded in interoceptive processes. While core interoceptive sensitivity remains stable across adulthood, the way bodily signals are attended to, interpreted, and utilised in emotional cognition undergoes systematic change. In younger adulthood, tighter mind–body coupling allows bodily signals to flexibly support learning for low-arousal information (for example, neutral face–name pairs) but can disrupt memory for higher-arousing negative stimuli (for example, angry face–name pairs). In later life, emotional selectivity toward positive information coincides with reduced reliance on raw visceral input and greater weighting of higher-order interoceptive beliefs and insight. This developmental shift provides a physiological extension of SST (Carstensen, 1993, 2006), indicating that the positivity bias in ageing reflects not only motivational priorities but also age-related changes in how bodily signals are integrated into emotional memory. More broadly, these findings highlight interoception as a key mechanism linking physiological ageing to emotional goals and cognitive regulation across the lifespan.

## Figures and Tables

**Table 1:** Reliability of Interoceptive Measures.

	All Groups	Young Adults	Older Adults
**BPQ-VSF**	0.89	0.88	0.91
MAIA-2 subscales			
Noticing	0.78	0.65	0.85
Not Distracting	0.83	0.85	0.82
Not Worrying	0.80	0.78	0.81
Attention Regulation	0.88	0.87	0.90
Emotional Awareness	0.85	0.75	0.91
Self-Regulation	0.88	0.87	0.89
Body Listening	0.86	0.82	0.90
Trusting	0.92	0.92	0.89
IAcc measures *			
HCT	0.98	0.96	0.98
HDT	0.59	0.51	0.41

Note. BPQ-VSF = The Very Short Form Body Perception Questionnaire, IAcc = Interoceptive Accuracy, HCT = Heartbeat Counting Task, HDT = Heartbeat Discrimination Task. Reliability coefficients are Cronbach’s alpha unless marked with *, which represents split-half reliability (Spearman–Brown corrected).

## Data Availability

The raw data supporting the conclusions of this article will be made available by the authors on request.

## Supplementary Materials

The following supporting information can be downloaded at: https://www.mdpi.com/article/10.3390/bs16050672/s1, Table S1: Age Group Comparisons: Interoception, Emotional Learning, and Correlation Strengths, Table S2: Age Group Comparisons: Interoception, Emotional Recall, and Correlation Strengths, and Table S3: Age Group Comparisons: Interoception, Emotional Recognition, and Correlation Strengths.
