# Supplementary material for: Age Differences in the Relationship Between Interoception and Emotional Processing
Source: Behav Sci (Basel). 2026 Apr 29;16(5):672. doi: 10.3390/bs16050672 (PMC13203222; doi:10.3390/bs16050672)
Supplement: Supplementary file 1 [file behavsci-16-00672-s001.zip › behavsci-4137744-supplementary.pdf]

Supplementary Material

Table S1. *Age Group Comparisons: Interoception, Emotional Learning, and Correlation Strengths*

Interoception	Emotion	*Young r*	n	*Older r*	Fisher's Z
BPQ-VSF	Angry	.107	40	-.012	-.051
	Neutral	.469*	40	.160	0.07
	Happy	.088	40	.134	0.20
MAIA Noticing	Angry	.085	40	-.121	-0.89
	Neutral	.263	40	-.061	-1.42
	Happy	.228	40	-.281	-2.24[†]
MAIA Not Distracting	Angry	-.005	40	.096	0.44
	Neutral	.121	40	-.096	-0.94
	Happy	.228	40	-.073	-1.31
MAIA Not Worrying	Angry	-.294	40	-.066	1.02
	Neutral	-.237	40	.191	1.87[†]
	Happy	-.224	40	.084	1.34
MAIA Attention Regulation	Angry	.042	40	-.043	-0.37
	Neutral	.164	40	-.028	-0.83
	Happy	.031	40	-.159	-0.82
MAIA Emotional Awareness	Angry	.064	40	-.123	-0.81
	Neutral	.179	40	.007	-0.75
	Happy	.242	40	-.123	-1.59
MAIA Self-Regulation	Angry	-.221	40	-.082	0.61
	Neutral	-.042	40	-.104	-0.27
	Happy	-.134	40	-.177	-0.19
MAIA Body Listening	Angry	.219	40	-.092	-1.35
	Neutral	.260	40	-.046	-1.34
	Happy	.248	40	-.118	-1.6
MAIA Trusting	Angry	-.094	40	-.163	-0.30
	Neutral	.114	40	.212	.043
	Happy	-.014	40	-.136	-0.53
HCT	Angry	.280	40	.356*	0.36
	Neutral	-.095	40	.083	0.77
	Happy	.315	40	.193	-0.56
HDT	Angry	.212	40	.215	0.01
	Neutral	.072	40	.156	0.37
	Happy	.172	40	.091	-0.35
HCT II	Angry	.002	40	-.094	-0.41
	Neutral	-.230	40	-.182	0.22
	Happy	.070	40	-.126	-0.85
HDT II	Angry	-.178	40	.082	1.13
	Neutral	-.441*	40	-.113	1.55
	Happy	-.194	40	-.074	0.53

Note. r represents one tailed partial correlations between interoception and emotional learning accuracy, controlling for BMI. Fisher's Z indicates the group differences in these correlations.

BPQ-VSF = The Very Short Form Body Perception Questionnaire, MAIA = Multidimensional Assessment of

Interoceptive Awareness, HCT = Heartbeat Counting Task, HDT = Heartbeat Discrimination Task, II = Interoceptive Insight, n = number of participants

* denotes significance following Lazerlere & Mulaik correction, †p<.05 significance value for Fisher's Z transformations.

Table S2

Age Group Comparisons: Interoception, Emotional Recall, and Correlation Strengths

Interoception	Emotion	*Young r*	n	*Older r*	Fisher's Z
BPQ-VSF	Angry	.274	40	-.327	2.67†††
	Neutral	.204	40	-.172	1.64
	Happy	-.195	40	-.357	0.22
MAIA Noticing	Angry	.228	40	-.110	1.47
	Neutral	.076	40	-.123	0.86
	Happy	-.121	40	-.301	0.81
MAIA Not Distracting	Angry	.050	40	-.099	0.64
	Neutral	.287	40	-.089	1.65
	Happy	-.082	40	.016	-0.42
MAIA Not Worrying	Angry	-.048	40	.213	-1.14
	Neutral	-.007	40	-.111	0.45
	Happy	.061	40	.213	-0.67
MAIA Attention Regulation	Angry	.103	40	-.084	0.81
	Neutral	.146	40	-.197	1.49
	Happy	-.161	40	-.162	0
MAIA Emotional Awareness	Angry	-.039	40	-.050	0.05
	Neutral	.076	40	-.019	0.41
	Happy	-.288	40	-.160	-0.58
MAIA Self-Regulation	Angry	.109	40	-.061	0.73
	Neutral	-.049	40	-.101	0.22
	Happy	-.138	40	-.103	-0.15
MAIA Body Listening	Angry	.141	40	-.132	1.18
	Neutral	.240	40	-.042	1.23
	Happy	-.014	40	-.131	0.51
MAIA Trusting	Angry	-.035	40	.131	-0.72
	Neutral	-.036	40	.243	-1.22
	Happy	-.124	40	.087	-0.91
HCT	Angry	-.359*	40	.039	-1.78†
	Neutral	-.109	40	-.260	0.67
	Happy	-.035	40	-.051	0.07
HDT	Angry	.001	40	-.364*	1.65
	Neutral	.083	40	.145	-0.27
	Happy	.142	40	-.201	1.49
HCT II	Angry	-.342*	40	.131	-2.1†
	Neutral	-.248	40	.090	-1.48
	Happy	-.031	40	.320	-1.56
HDT II	Angry	-.068	40	.258	-1.43
	Neutral	-.214	40	-.028	-0.81
	Happy	-.182	40	-.050	-0.58

Note. r represents one tailed partial correlations between interoception and emotional recall accuracy, controlling for BMI. Fisher's Z indicates the group differences in these correlations. BPQ-VSF = The Very Short Form Body Perception Questionnaire, MAIA = Multidimensional Assessment of Interoceptive Awareness, HCT = Heartbeat Counting Task, HDT = Heartbeat Discrimination Task, II = Interoceptive Insight, n = number of participants

* denotes significance following Lazerlere & Mulaik correction, $^{\dagger}p<.05$, $^{\dagger\dagger\dagger}p<.001$ significance value for Fisher's Z transformations.

Table S3

Age Group Comparisons: Interoception, Emotional Recognition, and Correlation Strengths

Interoception	Emotion	Young r	n	Older r	Fisher's Z
BPQ-VSF	Angry	-.001	40	-.166	0.72
	Neutral	.261	40	-.269	2.34†
	Happy	-.132	40	-.311	0.81
MAIA Noticing	Angry	.000	40	-.092	0.40
	Neutral	.108	40	-.280	1.7*
	Happy	-.121	40	-.276	0.70
MAIA Not Distracting	Angry	.000	40	-.046	0.20
	Neutral	.287	40	.017	1.20
	Happy	-.082	40	-.072	-0.04
MAIA Not Worrying	Angry	.021	40	.335	-1.41
	Neutral	-.007	40	.005	-0.05
	Happy	.061	40	.192	-0.57
MAIA Attention Regulation	Angry	-.033	40	-.190	0.69
	Neutral	.146	40	-.380	2.35†
	Happy	-.161	40	-.221	0.27
MAIA Emotional Awareness	Angry	-108	40	-.127	0.08
	Neutral	.076	40	-.300	1.66
	Happy	-.288	40	-.238	-0.23
MAIA Self-Regulation	Angry	.040	40	-.152	0.83
	Neutral	-.049	40	-.185	0.59
	Happy	-.138	40	-.105	-0.14
MAIA Body Listening	Angry	.133	40	-.259	1.72†
	Neutral	.240	40	-.230	2.06†
	Happy	-.014	40	-.167	0.66
MAIA Trusting	Angry	-.201	40	.126	-1.42
	Neutral	-.036	40	.060	-0.41
	Happy	-.124	40	.166	-1.26
HCT	Angry	-.028	40	.028	-0.24
	Neutral	-.109	40	-.178	0.30
	Happy	-.035	40	-.042	0.03
HDT	Angry	.191	40	-.407*	2.69†
	Neutral	.083	40	-.104	0.81
	Happy	.142	40	-.082	0.97
HCT II	Angry	-.102	40	.230	-1.45
	Neutral	-.255	40	.194	-1.97†
	Happy	-.165	40	.296	-2.03†
HDT II	Angry	-.230	40	.151	-1.66
	Neutral	-.306	40	.034	-1.51
	Happy	-.155	40	.135	-1.26

Note. r represents one tailed partial correlations between interoception and emotional recognition accuracy, controlling for BMI. Fisher's Z indicates the group differences in these correlations. BPQ-VSF = The Very Short Form Body Perception Questionnaire, MAIA = Multidimensional Assessment of Interoceptive Awareness, HCT = Heartbeat Counting Task, HDT = Heartbeat Discrimination Task, II = Interoceptive Insight, n = number of participants

* denotes significance following Lazerlere & Mulaik correction, [†]p<.05 significance value for Fisher's Z transformations.
